# (Pro)renin Receptor Is Present in Human Sperm and It Adversely Affects Sperm Fertility Ability

**DOI:** 10.3390/ijms22063215

**Published:** 2021-03-22

**Authors:** Marta Gianzo, Itziar Urizar-Arenaza, Iraia Muñoa-Hoyos, Zaloa Larreategui, Nicolás Garrido, Jon Irazusta, Nerea Subirán

**Affiliations:** 1 Department of Physiology, Faculty of Medicine and Nursing, University of the Basque Country (UPV/EHU), 48940 Bizkaia, Spain; itziar.urizara@ehu.eus (I.U.-A.); iraia.munoa@ehu.eus (I.M.-H.); jon.irazusta@ehu.eus (J.I.); 2 Innovation in Assisted Reproduction Group, Biocruces-Bizkaia Health Research Institute, 48903 Barakaldo, Spain; 3 Valencian Institute of Infertility (IVI-RMA)-Bilbao, 48940 Bizkaia, Spain; zaloa.larreategui@ivirma.com; 4 IVI Foundation, Instituto de Investigación Sanitaria La Fe (IIS La Fe), 46015 Valencia, Spain; Nicolas.Garrido@ivirma.com; 5 Research and Development Department, MEPRO Medical Reproductive Solutions, 20009 San Sebastian, Spain

**Keywords:** prorenin receptor, human, semen analysis, reproductive techniques, assisted, embryonic development

## Abstract

Sperm fertility ability may be modulated by different molecular systems, such as the renin-angiotensin system (RAS). Although renin is one of its most relevant peptides, the presence and role of the (pro)renin receptor (PRR) is completely unknown. We have proved for the first time the existence of PRR and its transcript in human sperm by western blot and RT-PCR. Immunofluorescence studies showed that this receptor is mainly located in the apical region over the acrosome and in the postacrosomal region of the sperm head and along the sperm tail. In addition, this prospective cohort study also proves that semen samples with higher percentages of PRR-positive spermatozoa are associated with poor sperm motility, worse blastocyst development and no-viable blastocysts. Our results provide insight into how PRR play a negative role in sperm physiology that it may condition human embryo quality and development. An in-depth understanding of the role of PRR in sperm fertility can help elucidate its role in male infertility, as well as establish biomarkers for the diagnosis or selection of sperm to use during assisted reproductive techniques.

## 1. Introduction

Currently, over 186 million people worldwide have infertility problems [1], and male factor represents 30 to 50 % of clinical infertility cases [2]. Abnormal sperm parameters including low sperm concentration and poor sperm motility contribute to fertility problems [3]. However, 20 to 30 % of men with normal parameters have fertility problems since they are unable to achieve pregnancy [4], suggesting that molecular deficiencies not described yet could cause male infertility [5]. Over the last few years, several studies have suggested that sperm molecular features, such as RNA and proteins, are involved in fertilization and embryo development [3,5,6,7,8]. A better comprehension of those sperm molecular characteristics will allow us to develop functionally relevant diagnostic and sperm selection strategies to maximize reproductive success [9].

Different communication systems such as opioids [10], tachykinins [11] or the renin-angiotensin system (RAS) [12,13,14] seem to be especially relevant in the regulation of sperm fertility. RAS is a communication system with endocrine characteristics, whose main role is to maintain blood pressure and the homeostasis of electrolytes and fluids. In addition, RAS components can also locally synthesize in multiple tissues and cells suggesting that this physiological system also exerts different actions at the local level [15]. Angiotensin II, angiotensin converting enzyme (ACE) and renin are the best-known active components of the RAS [15]. In this sense, although Ang II and renin are not synthesized in sperm [16], elevated levels of these proteins have been described in seminal plasma, which modulate different sperm functions. For its part, sperm ACE (tACE) is involved in motility, capacitation and acrosome reaction, and has been proposed as an essential protein in the fusion process of sperm and oocytes, as it interacts with oocyte zona pellucida (ZP). In fact, the aberrant, reduced or absent expression of tACE underlies failed fertilization, while fertility is rescued when the functional tACE gene is reintroduced. Likewise, it has also been associated with embryonic quality and development [13,14,17]. Although these peptides have traditionally been considered as the main bioactive molecules of the RAS, currently several studies have found new components of RAS that can activate different signal transduction pathways [15,18,19]. The (pro)renin receptor (PRR), also called ATP6AP2, is a recently reported component of the RAS. This transmembrane protein that can be activated directly by prorenin (the precursor of the renin protein) and by the renin itself [15,20]. One of the peculiarities of this receptor is that it is a main component of one of the new axes of the RAS recently described, the prorenin-renin-PRR axis [21]. It has been reported that the activation of PRR induces the lysis of angiotensinogen and, therefore, the synthesis of angiotensin I is important for several processes, such as: (1) the activation of H^+^-ATPase, (2) the formation of the Wnt receptor complex, and/or (3) the regulation of cell homeostasis and autophagy [15,20,21]. 

In male reproductive tissues, renin has been found in the epididymis, testes and seminal plasma [22], with higher levels of prorenin and renin in the latter than in blood plasma [23]. Furthermore, it has been suggested that there is an association between the prorenin levels of the seminal fluid and the quality of the semen [24]. However, there is no direct evidence of the presence of PRR in sperm cells. Therefore, the objective of this study was to determine whether PRR is present in human sperm. Once this first objective was verified, its association with basic sperm parameters, oocyte fertilization, embryonic development and reproductive success after in vitro fertilization (IVF) techniques was evaluated, in order to elucidate its potential value as a sperm biomarker for improve assisted reproduction techniques (ART).

## 2. Results

### 2.1. Patients and Seminal Samples 

The mean age of the 97 men whose semen samples were used in this study was 39.39 ± 0.45 years, ranging between 32 and 52 years. The seminal characteristics of the fresh semen samples and after preparation for in vitro fertilization techniques are indicated in Table 1. 

### 2.2. Expression and Localization of PRR in Human Sperm

**Figure 1:**
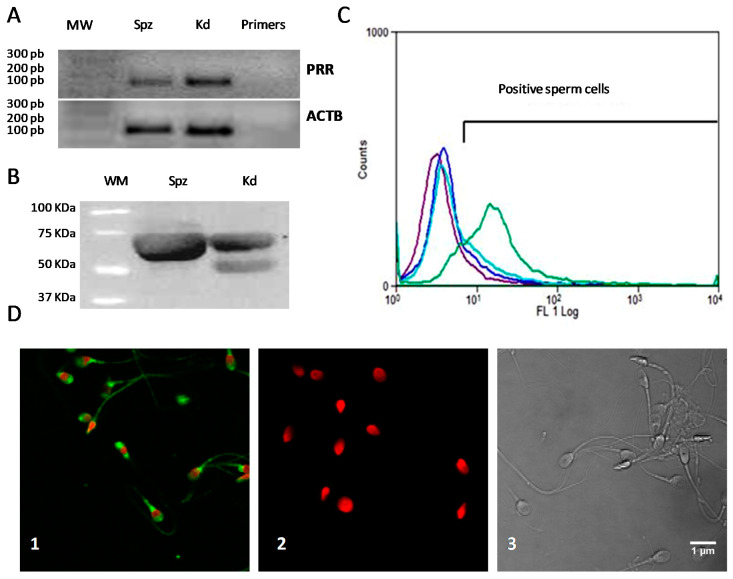
Expression and localization of PRR in human sperm cells. (**A**) A representative of midori-green-stained gels of the RT-PCR products for *ATP6AP2* and *ACTB* in human sperm from a pool of five normozoospermic semen samples (Spz) and kidney (Kd). Primers without cDNA were used as negative control. (**B**) A representative immmunoblot of PRR in human sperm from a pool of five normozoospermic samples (Spz) and kidney (kd). (**C**) A representative plot of PRR-positive sperm population analyzed by flow cytometry. Plots of blank control (purple) without both primary and secondary antibodies, negative primary antibody control (light blue) as the same concentration as the primary antibody and with secondary antibody, negative secondary antibody control (deep blue) with secondary antibody alone, and plot of PRR-positive sperm population (green). Nucleuses were stained with Hoescht 33258, *n* = 2. (**D**) Immunofluorescence analysis of the PRR in human sperm cells. (**D1**) PRR Positive sperm cells (green). (**D2**) Negative secondary antibody control (red). (**D3**) Phase-contrast image of the human sperm cells. Nucleuses were stained with Hoescht 33258. Representative photomicrographs are shown; *n* = 3. Scale bar: 1 µm.

To study the PRR gene expression, we analyzed the presence of the *ATP6AP2* transcript in human sperm cells by RT-PCR (Figure 1A). We detected the expected 151-bp fragment in human sperm samples and in the human renal cell line, which served as a positive control, thus confirming that *ATP6AP2* mRNA was present in human spermatozoa. The absence of amplicons in negative controls—omitting cDNA—confirms the absence of genomic DNA in each sample (Figure 1A).

Next, we described the presence of PRR protein by immunoblotting. Figure 1B shows a representative western blot using human sperm cells and human kidney, used as a positive control. The anti-PRR polyclonal antibody showed immunoreactivity at 56 kDa in human sperm protein extracts. In the kidney tissue, however, we detected two bands of 50 kDa and 70 KDa. No immunoreactivity was observed when the primary antibody was omitted before secondary antibody addition. Flow cytometry analyses confirmed the presence of PRR in human spermatozoa and the 68.99 ± 1.75% of cells were positive for PRR (Figure 1C). To assure that the fluorescence came only from spermatozoa, PRR signal was only measured in Hoechst-positive cells. The specificity of the primary antibody was confirmed using non-specific rabbit immunoglobulin fraction at the same concentration as the primary antibody. Secondary antibody specificity was evaluated by omitting the primary antibody before secondary antibody addition (Figure 1C). 

Finally, we also described the location of PRR in human spermatozoa by immunofluorescence approaches. Immunocytochemistry studies confirmed the presence of PRR in human sperm cells (Figure 1D). We observed a strong immunoreactivity mainly at the apical region over the acrosome and at the postacrosomal region of the sperm head. Moreover, we also observed a weak immunoreactivity along the sperm tail (Figure 1D). Cumulatively, 70% of cells were positive for PPR, confirming the results obtained by flow cytometry. Primary antibody specificity was confirmed by flow cytometry analysis. Fluorescent staining pattern was not evidenced after omitting the primary anti-PRR antibody before secondary antibody addition (Figure 1D). 

### 2.3. Correlation between PRR with Basic Sperm Parameters 

We analyzed the relationship between PRR and basic seminal parameters (concentration and motility) defined by the WHO [25]. The percentage of PRR-positive spermatozoa was negatively correlated with the semen concentration and total sperm count (Table 2). Regarding sperm motility, we observed a significant positive correlation between the percentage of PRR-positive cells and the percentage of NP spermatozoa.

### 2.4. Percentage of PRR-Positive Sperm Cells and Fertilization and Embryo Quality

The total number of donated oocytes was 1038 and the mean was 10.70 ± 0.32 oocytes received per donor. The fertilization rate of all cycles was 72.74% (70.34 ± 1.88%). 755 embryos were analyzed, with a mean of 7.80 ± 0.28 embryos per couple. 

In this prospective cohort study, we aimed to investigate the association between PRR-positive sperm cells and fertilization rate and intracitoplasmatic sperm injection (ICSI) outcomes in the cohort of zygotes obtained per recipient, although, for inherent reasons to the study, we cannot be sure whether the injected sperm was PRR positive or negative. 

First, we did not find any statistically significant correlation between PRR-positive sperm and fertilization rates. We next studied the relationship between late or early embryos and the percentage of PRR-positive sperm cells (measured at surplus sperm after IVF procedures). The mean characteristics of our embryo cohort were as follows (mean ± SEM): number of blastomeres on day two, 3.60 ± 0.05; embryo fragmentation on day two, 2.30 ± 0.15%; number of blastomeres on day three, 6.55 ± 0.08; and finally, embryo fragmentation on day three, 2.88 ± 0.17%. Concerning early embryo quality, we did not find any significant difference between the percentage of PRR-positive spermatozoa and embryo quality grades (*p* > 0.05) or embryo quality parameters (Table 3) on days two and three of development.

**Figure 2:**
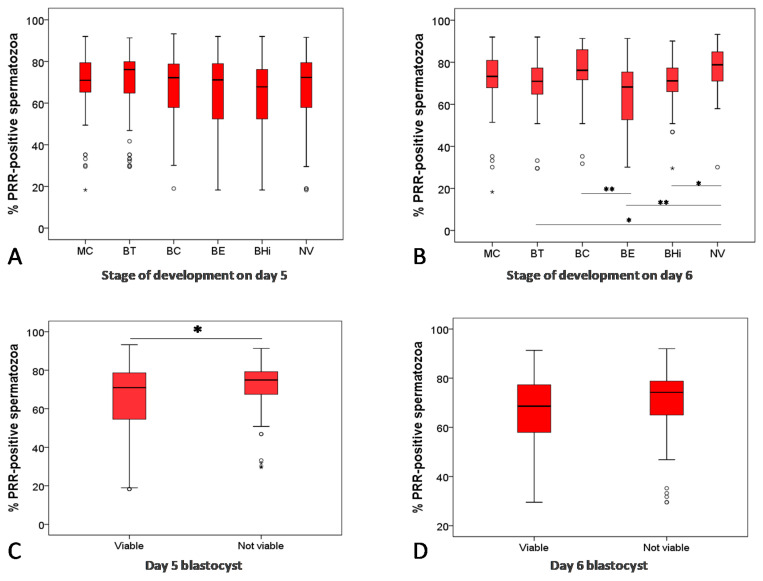
Graphic representation of the scoring and viability of the embryo on day 5 and day 6 and the association with the percentage of PRR-positive spermatozoa. Percentage of PRR-positive spermatozoa in association with the degree of development of the embryos on days 5 (**A**) and day 6 (**B**). Statistical differences among all groups were evaluated by using the Kruskall–Wallis test, followed by the Mann–Whitney *U*-test between two groups. MC: compact morula stage; BT: early blastocyst; BC: expanding blastocyst; BE: expanded blastocyst; BHi: hatching/hatched blastocyst; BD: blocked and degenerated. Blastocyst viability of embryos on day 5 (**C**) and day 6 (**D**). Statistical differences were examined between both groups by using the Mann–Whitney *U*-test. * Significant difference between groups (*p* < 0.05); ** high significant difference between groups (*p* < 0.01).

Considering that blastocysts have a higher implantation potential than early embryos, we also analyzed the association between PRR and the developmental stage of embryos in the later phase of in vitro development (Figure 2A,B). We observed that blocked or degenerated blastocysts (BD) were related to semen samples with higher levels of PRR-positive spermatozoa than early (BT), expanded (BE) and hatching/hatched (BHi) blastocysts (Figure 2B). 

To evaluate the relationship between PRR and blastocyst viability, we classified embryos into two groups: (1) embryos at the blastocyst stage were considered viable (V); and (2) arrested, degenerated, and blastocysts with lower development were considered non-viable (NV) (Figure 2C,D). Approximately 84% of the analyzed blastocysts were classified as viable. NV human blastocyst also came from sperm samples with higher percentages of PRR-positive spermatozoa (*p* < 0.05) (Figure 2C). 

### 2.5. Percentage of PRR-Positive Sperm Cells and Embryo Transfer, Clinical Pregnancy and Live-Birth Outcomes

Finally, we aimed to determine the relationship between sperm PRR and reproductive success. The mean characteristics of the ART outcomes were as follows (mean ± SEM): the number of embryos transferred per patient was 1.47 ± 0.06, the implantation rate was 52.25 ± 4.72, the mean frozen embryos per patient was 2.65 ± 0.25, and the number of viable embryos (transferred and cryopreserved) per patient was 4.15 ± 0.27.

For the evaluation of the association of PRR and reproductive success, we only used the first embryo transfer. A total of 89 of 97 cycles ended in embryo transfer (91.75% of those included in the study). Of all the transfers carried out, 63 resulted in biochemical pregnancies (70.78%), and later, 55 clinical pregnancies (61.79%) were confirmed by ultrasound. Finally, 49 cycles ended in a live birth (55.06% of the cycles with embryo transfer). However, when we analyzed the relationship between these reproductive outcomes with the percentage of PRR-positive spermatozoa, we did not find any statistically significant relationships (Table 4). 

## 3. Discussion

Sperm functions and subsequent embryonic development are complex processes that require tight regulation. In this regard, several cell communication systems are involved in the regulation of human sperm fertility capacity [10,11], including RAS [12,13,14]. One of the best-known components of the RAS is renin, an enzyme present in high levels in seminal plasma that seems to be involved in sperm density and motility [24]. In addition, different studies have also proved the involvement of tACE in sperm motility, capacitation, the acrosome reaction and sperm-oocyte fusion [14]. The present work has described for the first time the presence of PRR in human spermatozoa at the gene and protein level providing new evidence of the prorenin-renin/PRR axis in human spermatozoa. 

RT-PCR assays revealed the presence of the *ATP6AP2* in human spermatozoa. It has been suggested that a limited pool of RNAs is selectively retained and protected from degradation in mature sperm [26]. Those retained RNAs could play several roles in subsequent fertilization steps or in adequate embryo formation [27,28]. Hence, the presence of the PRR transcript can be important for mature sperm cells to be subsequently translated into protein upon fertilization [29]. However, mechanisms involving mRNA storage and silencing of the transcription, as well as the functionality of the mRNA in mature spermatozoa, is still a controversial issue that remains poorly understood [30].

Furthermore, this study has demonstrated the presence of PRR at the protein level in mature human spermatozoa. A unique band of 56 kDa of PRR protein was detected in human spermatozoa. Although the predicted molecular weight of this transmembrane protein is 45 kDa [15], several studies show a disparity in the size of the bands observed in different cell types ranging from 28 to 74 kDa [31,32,33], suggesting the presence of different isoforms of this receptor [34]. In addition, it has been reported that human sperm cells present different isoform proteins compared to other cell types [35]. Indirect immunofluorescence and flow cytometry analyses also confirm the presence of PRR on 70% of human spermatozoa. Specifically, immunoreactivity was detected at the apical region over the acrosome, at the postacrosomal region of the sperm head, and along the sperm tail. Given these data, PRR could be involved in the regulation of essential functions in sperm, such as motility, chemotaxis, sperm capacitation and/or acrosome reaction [35]. 

Basic semen analysis is the only routine clinical method to study male fertility status. This morpho-functional analysis, however, is unable to predict the fertility potential and reproductive outcome [4]. Male infertility is caused by a wide variety of undescribed deficiencies; hence, there is a need to improve and establish robust sperm quality indicators [36]. Over the years, several studies have identified a large number of sperm molecular characteristics, such as receptors, ion channels, mRNA or proteins, that are involved in sperm fertility and/or in embryo development [3,4,5,6,7,8,10,11,12,36]. Considering this, our next aim was to investigate the association between PRR levels and basic sperm parameters, human embryo development and reproductive success.

First, we evaluated the relationship between the PRR-positive cells with basic semen parameters. Flow cytometry results showed that the presence of PRR has a negative effect on sperm quality. Other membrane surface proteins are also negatively involved in sperm motility such as membrane antigen CD52 [37], neprilysin (NEP) [38], μ-opioid receptor (MOR) [39] or testicular isoform of angiotensin converting enzyme (tACE) [13]. Taking into account that sperm motility appears to be essential for natural reproduction, currently, the motility assay is the most reliable predictor of male factor infertility [12,37,40]. Based on our results, PRR could negatively affect the sperm fertilizing capacity. 

Additionally, the reproductive capacity of sperm is not only determined by its fertilizing potential, but also implies the first stages of embryonic development. Successful embryo development and subsequent pregnancy outcome are also linked to good quality semen samples [9]. Owing to this reason, our next purpose was to investigate the relationship between the percentage of sperm PRR with oocyte fertilization rates, embryo quality parameters and the reproductive outcome during ART. To fulfill this objective, we analyzed embryos from oocyte donation programs in order to avoid introducing bias concerning oocyte quality, letting us study the sperm features without the wide heterogeneity of the female factor [36]. Our study possesses some characteristics that reinforce the results obtained: (1) sperm PRR was determined in the same sperm aliquot that was used for assisted reproduction procedures, and (2) a large number of samples were included (*n* = 97). Regarding the fertilization rates, despite a negative relationship noticed between PRR and sperm motility, we did not observe any relationship between the PRR levels and fertilization rates. However, this could be because all cycles included in this study were inseminated by using intracitoplasmatic sperm injection (ICSI), in which the embryologist has bypassed the previous steps that take place in the natural conception, such as reaching the fertilization site and recognizing and fusing with the oocytes [9]. 

Since a sperm contribution may extend beyond fertilization, highlighting the fact that early and late paternal effects may be determinants of normal embryo development [7], we evaluated the relationship between PRR levels with embryo quality. Semen samples with higher percentages of PRR-positive spermatozoa are associated with worse blastocyst development. We found that blocked and degenerated blastocyst came from semen samples with higher levels of sperm PRR. In the later in vitro phase of development, thus, blastocysts with a higher implantation potential, such as expanded and hatching/hatched blastocysts [41], are associated with lower percentages of PRR-positive spermatozoa. Recent studies have reported that the PRR expressed during the embryo development plays an important role during embryo development and cell survival or viability [34,42,43]. In this regard, our results suggest that not only the embryo expressed PRR but also the spermatozoa expressed PRR can be involved in human embryo development. 

Previous studies report that PRR can play a key role in the invasion of trophectoderm (TE) cells in the maternal uterus, which is essential for the embryo implantation process. Nevertheless, we did not find any relation between the embryo transfer rate, clinical pregnancy and live birth with PRR levels. However, we cannot deny a possible effect of this receptor on the studied parameters because we only analyzed the first embryo transfer, where only the high-quality embryos were transferred. In consequence, this positive selection could be a factor of bias in our analysis, as have been described previously [36,44]. 

In brief, the present study describes for the first time the presence of PRR in human spermatozoa at the gene and protein level, providing new findings about the existence of the different axes of RAS in human sperm cells. The presence of different components of RAS in human spermatozoa can provide evidence of a relevant role of this system in sperm physiology. Specifically, higher levels of PRR in semen samples are associated with low sperm quality, blocked and/or degenerated embryos, and defective blastocyst development. Our results, thus, are consistent with the fact that low embryo quality and poor blastocyst development are associated with deficiencies in mature spermatozoa [40]. In fact, the obtained results indicate that not only may failures at the chromatin or mRNA level [26] compromise embryonic development, but sperm proteins can also be important for correct embryo development, as has been previously reported [40]. 

Due to the ethical implications related to the generation and use of human embryos in research, as well as the characteristics inherent to the microinjection technique itself, the data shown do not allow us to ensure whether the injected spermatozoon is PRR positive or negative. However, the results shown in this study reflect the association of the PRR present in human spermatozoa, since surplus aliquots of samples used in assisted reproduction treatments have been used for its analysis. 

Our data reinforces the sperm role in embryo development, changing the old idea of sperm as a mere “DNA vehicle carrier” [7,36]. These clinical observations led to the hypothesis that PRR plays a negative role in sperm fertility, suggesting that PRR could be considered as a potential marker of sperm quality. This negative association with embryo health appears quite promising in predicting the quality of the embryo after in vitro procedures. That is, why these data could serve as a first step to establish future studies that show more directly and reliably the role of PRR of sperm for this purpose. All these observations contribute to the hypothesis that sperm PRR could be used as a tool for the negative selection of the most appropriate sperm for assisted reproduction techniques.

## 4. Materials and Methods 

### 4.1. Ethical Approval

Semen samples were obtained from male partners of couples who underwent IVF treatments with donated oocytes at the Clínica IVI Bilbao, Basque Country, Spain. Study participation and sperm samples for research were obtained after written consent from patients. In order not to intervene or harm any of the reproductive treatments evaluated in this study, the aliquots of the sperm samples used in the molecular analyzes were collected once the microinjection process was completed. All samples and data were kept anonymous. All experiments were performed in accordance with relevant guidelines and regulations. 

### 4.2. Patients and Semen Analysis

A total of 120 patients were included between February 2014 and July 2015. A total of 23 normozoospermic samples were used to determine the presence and location of the PRR in human spermatozoa and 97 semen samples were analyzed to elucidate the implication of PRR in male fertility status and in embryo development. 

Ejaculates were collected into sterile containers by masturbation on site, after a two-to-five-day period of sexual abstinence, the day of oocyte retrieval and, after retrieval allowed to liquefy at 37 °C and 5% (*v*/*v*) CO_2_ for 10 min before processing. Semen volume, as well as sperm concentration and motility, were measured for each sample in duplicate, in a Mackler® Chamber (Sefi Laboratories, Haifa, Israel). According to World Health Organization (WHO) guidelines [25], sperm motility was classified into three different groups: (1) spermatozoa with progressive motility (PR), (2) non-progressive motility (NP), and (3) non-motile spermatozoa (IM). 

Subsequently, semen samples were centrifuged at 300 g for 20 min through a discontinuous colloidal silica density gradient (45 to 90%) of PureSperm (Nidacon, Gothenberg, Sweden). The pellets were then collected and washed (at 400 g for 5 min) in 2 mL of culture medium Global® for fertilization Medium (Life Global Group, Brussels, Belgium) and spermatozoa were re-suspended in 0.2 to 1 mL of medium for IVF techniques. After this, the concentration and mobility of the obtained sperm samples were measured again.

Surplus spermatozoa remaining after being used for the ICSI technique were then collected for molecular analysis by flow cytometry. The molecular data obtained were related to basic sperm parameters (measured in fresh samples, as well as after being processed), embryo quality parameters and reproductive outcomes.

### 4.3. Reverse Transcription-Polymerase Chain Reaction Analysis

The RNAs of spermatozoa and the human renal cell line (RC-124), used as a positive control, were isolated with the Nucleo Spin RNA II kit (Macherey-Nagel, Düren, Germany), including a DNase digestion step using an RNase-free DNase kit (QIAGEN, Limburgo, Netherlands) to exclude possible contamination by genomic DNA. First-strand cDNA synthesis was conducted using Superscript II RT (Invitrogen, LifeTechnology, Carlsbad, CA, EEUU) according to the manufacturer’s instructions using 600 ng of RNA. Reverse transcription-polymerase chain reaction (RT-PCR) was performed using SYBR® green master mix (LifeTechnology, Carlsbad, CA, EEUU) with 2.5 µL of diluted cDNA and 0.25 µM primers per 12.5 µL per reaction on an ABIPrism 7000 sequence detection system (Applied Biosystems, LifeTechnology, Carlsbad, CA, EEUU). Specific oligonucleotide primer pairs used for PCR were the following: human PRR: forward primer, 5-TGGTAGGGAAGGCAAACTCA-3, and reverse primer, 5-TCAGAAAGAAAGAGCAGGTCAA-3; and human ACTB (β-actin): forward primer, 5-GGCACCCAGCACAATGAAG-3, and reverse primer, 5-CCGATCCACACGGAGTACTTG-3, used as an internal control. These primers were designed based on cDNA sequence. PCRs were performed using the following parameters: 25 °C for 10 min, 42 °C for 50 min followed by a final extension step at 70 °C for 15 min. Analysis was carried out on samples measured in triplicates. The RT-PCR products were also separated by 1.5% agarose gel electrophoresis. The amplicon sizes were verified by comparison with a DNA size ladder.

### 4.4. Western Blotting

Isolated spermatozoa and human kidney samples were resuspended in modified lysis buffer (radio-immunoprecipitation assay (RIPA) buffer (Sigma-Aldrich, Saint Luis, MO, USA), protease and phosphatase inhibitor cocktail (Sigma-Aldrich, Saint Luis, MO, USA) and DNase (Quiagen, Limburgo, Netherlands)) to obtain protein extracts. The sperm samples were sonicated (two times 70% amplitude, 0.5 s 15 burst, and one time 40% amplitude, 0.5 s 15 burst), and incubated for 30 min on ice. Human kidney, used as a positive control, was minced with sharp scissors, suspended in lysis buffer and homogenized (Teflon glass), and then was incubated for 1 h on ice. The homogenates were centrifuged at 18,800 *g* for 15 min at 4 °C, and the clear supernatants were then used for the western blot studies. Protein concentrations were measured by Bicinchonicic acid method (Sigma-Aldrich, San Luis, USA). 

The protein extracts were diluted in Laemmli sample buffer containing β-mercaptoethanol (5% *v*/*v*) and boiled for 5 min. Proteins (50 µg sperm protein; 30 µg kidney protein) were loaded onto 10% resolving gels, separated by one-dimensional SDS-PAGE and then transferred to polyvinylidene fluoride (PVDF) membranes (Amersham Hybond^TM^, GE Healthcare Bio-Sciences K. K. Tokyo, Japan) using the Mini Trans- Blot electrophoretic transfer system (Bio-Rad Laboratories, Hercules, CA, USA). After transfer, each membrane was blocked with Blotto (20 mM Tris-HCl, pH 7.5, 0.15 M NaCl, 1% Triton X-100) containing 5% nonfat dry milk (blocking buffer) for 1 h and then incubated with a dilution of polyclonal rabbit anti-renin receptor antibody (Renin receptor antibody [H-85], sc-67390; Santa Cruz Biotechnology, Inc., Heidelberg, Germany) at a dilution of 1:800 overnight at 4 °C. After washing (3 × 5 min) in Blotto buffer, the membrane was incubated for 1 h at room temperature with peroxidase-conjugated goat anti-rabbit IgG antibody at a dilution of 1:3000 (Goat anti-rabbit IgG HRP, ab6112; Abcam, Cambridge, UK). After washing (3 × 5 min) with Blotto buffer, the peroxidase activity of the transfer proteins was revealed by enhanced chemiluminescence (ECL).

### 4.5. Flow Cytometry

Spermatozoa were fixed with 4 % (*v*/*v*) paraformaldehyde (PAF) (Sigma-Aldrich, Saint Louis, MO, USA). Samples were permeabilized with 0.5% Triton X-100 (10 min), incubated in blocking medium (PBS with bovine fetal serum [BFS] (Biochrom, Cambridge, United Kingdom) 10% (*v*/*v*)) for 30 min. The primary antibody designed to recognize human PRR (renin receptor antibody [H-85], sc-67390; Santa Cruz Biotechnology, Inc., Heidelberg, Germany) was diluted 1:250 in PBS with BFS 5% and incubated overnight at 4 °C. Goat anti-rabbit IgG conjugated with Alexa Fluor 488 (Invitrogen, LifeTechnology, Carlsbad, CA, EEUU) secondary antibody was used at a 1:2000 dilution and nuclei were stained with 0.5 μg/mL Hoechst 33258 (Molecular Proves, Eugene, OR, USA). Primary antibody specificity was performed using a negative unspecific rabbit immunoglobulin fraction (normal) (Dako, Glostrup, Denmark) at the same concentration as the primary antibody, as described elsewhere [45]. Secondary antibody specificity was performed omitting the primary antibody before secondary antibody addition. The flow cytometry analysis was performed on a Gallios^TM^ flow cytometer (Becton Dickinson, San Jose, CA, USA). At least 10,000 events were analyzed per sample. Blue fluorescence (Hoechst 33258) and green florescence (Alexa Fluor 488) were collected in the FL9 and FL1 sensors, respectively. To exclude the debris from the analysis, we used a discrimination frame around the sperm population on the forward (FSC) and side (SSC) scatter plots and only selected the Hoechst 33258-positive events. Results of the Alexa Fluor 488 and Hoechst 33258 fluorescence were analyzed with the Summit v4.3 software.

### 4.6. Immunofluorescence 

To localize the PRR in spermatozoa, isolated sperm cells were fixed with 4% PAF and smeared onto a slide coated with poly-l-lysine. Triplicate slides were prepared for each sample. Samples were permeabilized in the presence of 0.5% Triton X-100 (10 min) and blocked in PBS and incubated for 30 min in PBS with FBS 10% (*v*/*v*). For indirect immunofluorescence staining, slides were incubated with anti-PRR (renin receptor antibody [H-85], sc-67390, Santa Cruz Biotechnology, Inc., Germany) at a dilution of 1:500 overnight at 4 °C. This antibody recognized an extracellular domain of renin receptor of human origin. Slides were then washed in PBS three times, incubated with Alexa Fluor 488 goat anti-rabbit IgG secondary antibody (1:1000) (Molecular Probes, Eugene, OR, USA) for 1 h at room temperature in the absence of light and subsequently, washed in PBS three times. We stained the nuclei with Hoechst 33258 at 2 min. Finally, the slides were mounted using Fluoromount G reagent (Southern Biotech, Birmingham, AL USA), and examined by confocal microscopy (Olympus Fluoview FV 500, Olympus. Tokio, Japan). Negative controls were performed in the same way, except for the omission of the primary antibody before secondary antibody addition. 

### 4.7. Assisted Reproductive Technology Procedures

The employment of an oocyte donation model reduces the variability of oocyte quality associated with female infertility and reduces endometrial receptivity variability associated with ovarian stimulation protocols. Therefore, it allowed us to analyze the relationship between PRR-positive spermatozoa and fertilization rates, embryo quality and development, implantation rates, and reproductive outcomes controlling the potential bias caused by female factor [36]. Recruitment and management protocols for oocyte donors, as well as the steroid replacement on the recipients have been previously described [46]. Recovered mature oocytes (metaphase II) were inseminated by means of ICSI after the protocol that was described elsewhere [47]. Subsequently, microinjected oocytes were cultured in a normal Petri dishes (Falcon, Welwyn, UK) in 20 mL drops of culture media (Cleavage Medium; Cook Medical IVF, Limerick, Ireland) under mineral oil. After fertilization checking, zygotes were placed individually in a new pre-equilibrated Petri dish with drops of 50 mL of the same culture medium covered with 7 mL of mineral oil. On the basis of previous medical criteria, all patients were assigned a day of embryo transference (day 3 vs. day 5 or 6); depending on that clinical decision, embryos were cultured until embryo transfer at day 3 in Cleavage Medium (Cook Medical IVF), and from day 3 to day 5 in CCM Medium (Vitrolife, Göteborg, Sweden). Both, microinjected oocytes and embryos were cultured at 37 °C in a controlled atmosphere of CO_2_ at 5% (*v*/*v*) in a conventional incubator (Heraeus; Heracell, Hanau, Alemania).

### 4.8. Fertilization Rates, Embryo Quality, Transference, Biochemical and Clinical Pregnancy and Live-Birth Outcome

Successful fertilization was verified by confirmation of two polar bodies and two pronuclei 16 to 19 h after microinjection. Embryo quality was assessed at both the cleavage stage (days 2 and 3) or early embryos, and in the later phase of in vitro development (days 5 and 6), at the blastocyst stage. In both cases, the morphology of the embryos was evaluated at 40× magnification under an inverted microscope. Early embryos were categorized in four grades from A (high quality) to D (low quality) considering the number of blastomeres, their fragmentation, multinucleation and symmetry, the presence and number of vacuoles, and the appearance of the zona pellucida, according to the Spanish Association of Reproduction Biology Studies (ASEBIR) criteria, when grade A gave the best and grade D the worst prognosis for implantation for a combination of the various aforementioned morphologic parameters [48]. At day 2 (44 to 47 h postinsemination), grade A embryos have four cells, <11% fragmentation, even size and no multinucleation; grade B embryos have two or five cells with 11 to 25% fragmentation and no multinucleation; grade C embryos have two to six cells, 26 to 35% fragmentation, uneven cell allowed but no multinucleation; and grade D, if the embryo has one or greater than six cells, multinucleation, abundant vacuoles and >35% fragmentation or type IV fragmentation [48]. To classify the day 3 embryos (67 to 71 h postinsemination), all four categories were assigned according to the evolution of the embryos from day 2 to day 3 [49]. Grade A embryos have seven to eight cells, <10% fragmentation, even size and no multinucleation; grade B embryos have seven to eight cells with 11 to 25% fragmentation, nine evenly sized cells or more and no multinucleation; grade C embryos have less than six cells, 26 to 35% fragmentation, uneven cells allowed but no multinucleation; and grade D, if the embryo has six cells or less and observation of multinucleation, >35% fragmentation or type IV fragmentation [48]. All embryos were included in the determination of the patient’s mean embryo score. Embryo quality was related to sperm PRR in two different ways: as an individual concept by grouping the embryos according to quality or determining an embryo quality score per cohort. To assess the embryo quality score per cohort, each embryo grade was assigned a value (1, 2, 3, 4); grade A embryos were assigned a value of 1 and grade D a value of 4. Moreover, some of the early embryo parameters were calculated as an average of the embryo cohort per patient, including the fertilization rate, embryo fragmentation, average number of cells and symmetry. 

Human blastocysts were scored on day five and six of embryo development (114 to 118 and 136 to 140 h post-insemination, respectively). Blastocysts were grouped in four groups according to the expansion of the blastocoele cavity: early (BT), expanding (BC), expanded (BE), or hatching or hatched (BHi) blastocysts [49]. In addition, embryos with slower development that remained at the compact morula stage (MC), as well as embryos that were blocked or degenerated (BD) were also considered. To investigate the relationship between sperm PRR and blastocyst viability, blastocysts were classified as viable (V) if they were transferred or frozen, or non-viable (NV), if they were arrested or of poor quality. 

In each case, transfers were performed on day 3 or day 5 to 6 after microinjection, and only one or two good embryos were selected for transfer. Supernumerary embryos were frozen for eventual future transfers. Biochemical pregnancy was considered after the detection of a positive result in the β-hCG test (defined as having a serum level of β-hCG greater than 10 IU/mL) in the recipients 16 days after the donor’s oocyte retrieval. Clinical pregnancy was determined by observing a gestational sac with a fetal heartbeat at seven weeks of pregnancy detected by transvaginal ultrasound, and live-birth outcome when the cycle ended with an infant born alive. In estimating reproductive success, we only considered the first embryo transfer.

### 4.9. Statistical Analysis

The percentages of PRR-positive sperm cells were associated with basic sperm parameters, embryo quality parameters and reproductive success. The normality of the data was evaluated using the Kolmogorov–Smirnov test. Normal data distributions were not detected, so non-parametric tests were used. To analyze the correlation between the number of positive sperm cells for PRR and basic sperm parameters and embryo quality parameters, we used a scatter plot and Spearman´s rank correlation analyses. Differences between PRR-positive sperm cells and fertilization rates, late embryo development, blastocysts viability, embryo implantation, pregnancy outcomes and live-birth success were analyzed by Kruskal–Wallis and Mann–Whitney *U* tests. Statistical significance and high statistical significance were determined by *p* values (*p* < 0.05 and *p* < 0.01, correspondingly). Statistical analyses were performed using the IBM SPSS Statistics 22 software.

## Figures and Tables

**Table 1:** Seminal characteristics of fresh semen samples and after preparation for in vitro fertilization techniques.

Seminal Parameters	Fresh Samples	Processed Samples
Volume (mL)	2.99 ± 0.15	0.76 ± 0.02
Sperm concentration (×10^6^ spz/mL)	75.31 ± 4.05	14.86 ± 1.19
Total sperm count (×10^6^ spz)	205.94 ± 13.45	11.99 ± 1.13
Progressive motility (%)	54.42 ± 1.75	94.67 ± 0.49
Non-motile spermatozoa (%)	37.87 ± 1.49	4.54 ± 0.49

Results are expressed as mean ± SEM. Spz: spermatozoa.

**Table 2:** Correlations between PRR with human sperm fertility status.

			Fresh Samples	Processed Samples
Total sperm count		Correlation coefficient	0.043	−0.257 *
		*p* value	0.676	0.011
Concentration		Correlation coefficient	−0.091	−0.259 *
		*p* value	0.373	0.010
Motility	Progressive motility (PR)	Correlation coefficient	0.087	−0.081
		*p* value	0.399	0.428
	Non-progressive motility (NP)	Correlation coefficient	0.035	0.274 **
		*p* value	0.736	0.007
	Immotility (IM)	Correlation coefficient	−0.089	−0.025
		*p* value	0.384	0.811

Spearman rank correlation coefficients analyses among the percentage of PRR-positive spermatozoa in association with sperm samples concentration and the different types of motility. * Significant difference between groups (*p* < 0.05), and ** high significant difference between groups (*p* < 0.01).

**Table 3:** Correlations between PRR-positive sperm cells and fertilization rate and embryo quality.

	Correlation Coefficient	*p* Value
Fertilization Rate	−0.130	0.204
Day 2 embryo		
Embryo quality	0.061	0.558
Number of blastomeres	0.084	0.423
Embryo fragmentation	0.071	0.490
Embryo symmetry	0.050	0.627
Day 3 embryo		
Embryo quality	0.010	0.920
Number of blastomeres	0.053	0.605
Embryo fragmentation	0.118	0.257
Embryo symmetry	0.050	0.627

Spearman rank correlation coefficients analyses among the percentage of PRR-positive spermatozoa in association with fertilization rate, embryo quality, average number of cells, average embryo fragmentation, and average embryo symmetry on days 2 and 3 per patient.

**Table 4:** Association of sperm PRR levels with reproductive outcomes.

	Yes	No	*p*(UMW)
Embryo transfer	72.26 (*n* = 89)	69.71 (*n* = 8)	0.704
Biochemical pregnancy	71.93 (*n* = 63)	74.22 (*n* = 26)	0.427
Clinical pregnancy	71.46 (*n* = 55)	75.00 (*n* = 34)	0.303
Live-birth outcome	71.46 (*n* = 49)	74.22 (*n* = 40)	0.453

The table shows the median values of each group. *p*: bilateral signification of the non-parametric Mann–Whitney *U*-test analyses.

## Data Availability

The data presented in this study are available on request from the corresponding author.

## Abbreviations

ACE	Angiotensin Converting Enzyme
ACTB	Beta-actin
ART	Assisted Reproductive Techniques
ASEBIR	Spanish Association of Reproduction Biology Studies
BC	Expanding blastocysts
BD	Blocked or degenerated blastocysts
BE	Expanded blastocysts
BHi	Hatching or hatched blastocysts
BT	Early blastocysts
ICSI	Intracitoplasmatic sperm injection
IM	Non-motile spermatozoa
IVF	In vitro fertilization
MC	Morula stage
NP	Spermatozoa with non-progressive motility
NV	Non-viable blastocysts
PR	Spermatozoa with progressive motility
PRR	(Pro)renin receptor
RAS	Renin-angiotensin system
V	Viable blastocysts
WHO	World Health Organization
